# Hospital and Institutional News

**Published:** 1916-04-01

**Authors:** 


### HOSPITAL AND INSTITUTIONAL NEWS.

### THE ROYAL COLLEGES AND THE CENTRAL MEDICAL WAR COUNCIL.

After a good deal of spurring, the Royal Colleges of Physicians and of Surgeons have at last officially joined in the task of supplying the needs of the Royal Army Medical Corps for commissioned officers. To many members of the medical profession it has been inexplicable that the Colleges have been so slow to move; but recrimination will now, doubtless, be a thing of the past in view of the action at last taken, provided that action be genuinely helpful and efficient. It seems at the moment more than a little doubtful what the exact status and duties of the Colleges' representatives will be. All that is known definitely is that they are to have official standing, that they will work separately from the Central War Committee but towards the same goal, and that they will act as an Advisory Committee to assist the medical authorities at the War Office. It has been also stated that they will deal with cases affecting the Metropolitan hospitals and schools, whereas the Central Committee will deal with the country at large (Scotland retaining, as heretofore, control of her own arrangements). We should prefer to see the Royal Colleges taking a wider purview than this announcement would appear to indicate; though we admit that some scheme is necessary to prevent overlapping and clashing between the Central Committee and the Colleges, and that a delimitation of spheres of interest is perhaps necessary.

### MR. HUGHES AND WOUNDED AUSTRALIANS.

Among all the visits to war hospitals which have been lately paid none has excited more interest than that of Mr. Hughes, Prime Minister of Australia, to the Australian Auxiliary Hospital, Harefield. Patients, doctors, and nurses are all Australians, and eager, like the rest of us, to greet the Minister of the hour; for Mr. Hughes' vigorous speeches have certainly invigorated the country. He reminded the Australians of the gratitude of the Commonwealth; in fighting in this war, he added, they had done a great thing for Australia, but an even greater thing for civilisation. Colonel Giblin, Australian Army Medical Service, accompanied Mr. Hughes, who spent almost the whole day at the hospital.

### A CURIOUS POINT IN LAW.

Considerable prominence has been given in some of the daily papers to an incident which occurred recently at an inquest in London, when the Coroner refused to accept a second-hand account of a *post-mortem* examination, and insisted on the attendance of the surgeon who had made it. Owing to press of work in the hospital, the surgeon returned a message to the Court to say that he would not come, thereby laying himself open to summary penalty for contempt of Court. In due course he arrived and gave the required evidence, and the incident terminated. It elicited, however, a statement on the law of inquests from the Coroner which contained one curious and little-known point. Apart from committal by the Coroner himself for contempt, a defaulting witness may by common law be prosecuted before a Court of summary jurisdiction either by the Coroner himself or by any two of the jurors. Apparently this latter curious privilege of a Coroner's juryman has never been exercised; but it exists, and could be put into force if circumstances required it.

### AN ARCHITECT'S MUNIFICENCE.

The will of Lieut.-Colonel H. L. Florence, V.D., the architect, who died recently at Bath, contains many large bequests to charitable institutions, as well as to public bodies, such as the National Gallery, the Dulwich Gallery, and the Victoria and Albert Museum. Of these latter bequests, the most interesting institutionally is the picture of Greenwich Hospital by James Holland, which is left to the Trustees of the National Gallery. To hospitals some magnificent legacies are devoted: St. Bartholomew's and Charing Cross Hospitals get £10,000 apiece, as well as a share each in the residual estate (which reaches, it is stated, to nearly £100,000). The Foundling Hospital has a specific legacy of £250; and other hospitals will receive some day shares in the residual estate, which is subject to two life interests and cannot therefore be distributed at once.

### SCOTLAND'S FIRST OPEN-AIR HOSPITAL.

Sir Thomas Burnett, of Leys, chairman of the directors of the Royal Aberdeen Hospital for Sick Children, recently gave some information on the subject of hospital construction in Scotland. In alluding, at the annual meeting, to the scheme for the building of an open-air ward at Kepplestone, he said that there were two objects in view. The first was to increase accommodation, and thus to reduce the waiting list; and the second was to gain experience of open-air ward construction before erecting the new hospital at Ashley. Though, he added, open-air treatment had been found beneficial in England for the treatment of surgical as well as of consumptive cases, no open-air hospital had been built in Scotland. Only experience could show whether such treatment was equally practicable in the more rigorous climate of the Northern kingdom. If the new ward at Kepplestone proves a success, it will be removed to Ashley when the new buildings are erected there. Dr. Gordon, on the same occasion, declared that the difference in average temperature between the Midlands and South of England and the Northern land was not more than 1.5 or 2.0. But, he added, in the South and Midlands of England there would be from fifteen to twenty-six hours more sunshine per month. If Aberdeen were the first in Scotland to found an open-air hospital for children, it would be a proud day for the city.

### DENTAL SURGERY AT GUY'S HOSPITAL.

Mr. J. Bromley, Dean of Guy's Hospital, in appealing to the Southwark Tribunal for the exemption from military service of fourteen dental students, drew attention to the position of dental surgery at the present time. He pointed out that owing to the decrease of dental surgeons they had been compelled to close down part of the dental surgery at the hospital, and to discontinue the treatment of children suffering from deformity of the jaw. Most of the applicants for exemption were third-year students, and, if called upon to join the Army, they would have no opportunity of continuing their studies and practice. The War Office had granted a very limited number of dental commissions, and, as the dental treatment of the Forces was appalling, there was a great deal of dissatisfaction. They were hoping that more dental commissions would be granted as a result of the present agitation. The Tribunal, which has had to deal with a large number of applications for exemption from Guy's, adopted their usual policy of granting each student six months' extension.

### A LEAFLET ON MEASLES.

The Women's Imperial Health Association has prepared a leaflet on measles for the guidance and instruction of laymen, and more especially laywomen, in the leading facts about this disease. How the general impression got about amongst parents and guardians that measles is a negligible disease, which a child had best have as soon as possible, to "get it over," is hard to say: possibly partly by failure to discriminate between measles and roseola (unfortunately named "German measles" by some physician who has much to answer for), partly by comparison with the (former) terrors of diphtheria and scarlet fever, now minimised. At any rate, intelligent persons who read this leaflet are not likely to hold measles too cheaply; if anything, they may get slightly panic-stricken. The complications are set forth with refreshing frankness, and altogether it is made quite clear what doctors have long been preaching, that measles is a serious illness, and should be regarded as such. We think the leaflet should do much good, and that the Women's Imperial Health Association is to be commended for printing it.

### ANOTHER POOR-LAW INSTITUTION AS A MILITARY HOSPITAL.

At the request of the Local Government Board the Bermondsey Board of Guardians have offered to the War Council their Ladywell Institution for the purpose of being converted into a military hospital, to be known as the Bermondsey Military Hospital, Ladywell. The institution has been used hitherto as a home for the aged, and accommodates close on 1,000 people. It is situated at Lewisham, amidst the most delightful surroundings, and with large gardens attached. The inmates displaced are to be accommodated at the Tanner Street and Parish Street Institutions, and at St. Anne's Home, Streatham, which belongs to the St. Pancras Board of Guardians. For many months the Tanner Street Institution and St. Anne's Home have been used by the Belgian Consulate as hospitals for wounded and sick Belgian soldiers, but the Belgian Government has decided to relinquish these hospitals immediately after the birthday celebrations of King Albert on April 8, other arrangements having been made for the accommodation of his soldiers.

### MR. BAKER'S REWARD AT HERTFORD.

Success is always more popular than the vigour and resource on which it depends; so when we read of the congratulations which have attended the promising balance in hand recorded in the latest report of the Hertford County Hospital, we are reminded of a certain discussion which took place about a year ago. There was a cry for money; there was a growing annual deficit of some £700; and there was considerable doubt as to whether "it was any good" to do anything. Into this typical Slough of Despond Mr. H. Clinton Baker, the High Sheriff, and, if we mistake not, the Vice-Chairman, Admiral Hastings, dropped the twin stones of vigour and resource, and caused a very desirable troubling of the waters. As The Hospital no less vigorously backed them up, the successful year which has attended their efforts is as gratifying as it was inevitable. The timid question "Is it any good to do anything *now*?" is not the way to surmount difficulties. These always disappear before such a policy as that which Mr. Clinton Baker has pursued. But we hope that the critics of a year ago and the acclaimers of to-day will draw the moral—that when such difficulties recur Mr. Baker's method is the right one.

### THE PROSPERITY OF INDUSTRIAL TOWNS.

The annual report of the Leicester and County Saturday Hospital Society testifies at once to the prosperity of the industries of the county and to the continued interest of the workers. As the report states, "the Hospital Saturday Fund is firmly set in the habits of the people," with the result that the total contributions for the year amount to £16,449, an increase of £622 on the previous year. "When we realise that thousands of men (mostly contributors to the Society) have joined His Majesty's Forces, it is difficult to see how the increase is accounted for—the explanation is that a vast number of new workers have been absorbed in the staple trades, new industries have been introduced, and our system of contributions being so well established there has been no difficulty in adding the newcomers to the number of contributors." In order to consolidate the finances of the Society, £2,500 was invested in War Loan. The income of the Society consists mainly of three-tenths of the net income from workpeople's contributions; the remaining seven-tenths goes to the Royal Infirmary. The total income was £7,206, and the expenditure (including the War Loan investment of £2,500) was £7,056, so that a balance of £150 was carried forward. £2,600 was deposited at interest with the bankers. Grants were made to the following charities:—£42 to the Leicester District Nursing Association; £21 to the Royal Surgical Aid Society; £15 15s. to the Leicester Institution for Diseases of the Skin; £10 10s. to the Birmingham and Counties Sanatorium; and £5 5s. each to the Highfields Hospital and the Leicester Maternity Hospital.

### ECONOMY AT BIDEFORD.

At the recent annual meeting of the Bideford Hospital attention was drawn to the low expenditure on provisions during the year 1915, in spite of the general rise in prices. The total expenditure for this item was £282. The daily average number of patients boarded was 19.81—say 20; the nursing staff numbers five, and the domestic staff probably three, which makes a daily average of twenty-eight persons to be fed. Thus the average cost of food per head for patients and staff together works out at about 6¾d. per diem, or a trifle over £10 a year. We should have no hesitation in rejecting this as insufficient to meet the requirements of the household were it not for one gratifying circumstance mentioned at the meeting. The committee state that the numerous gifts of produce have been a considerable help to the supplies, and we congratulate the matron warmly both on the liberality she has been able to evoke in this direction, and on the admirable housekeeping skill which has enabled her to turn indiscriminate gifts to such good account. Another year some estimate might with advantage be attempted of the value to the institution of the gifts in kind contributed for the table of patients and staff. Such produce is often of the nature of luxuries—fruit, game, poultry, jams, etc.—and it is by no means an easy task to make it tell in reducing the weekly bills. Very much more would be given out of the surplus of large gardens and estates could the donors be made to understand, as at Bideford, that their generosity stood for the equivalent of large monetary donations in the finance of the year.

### THE CHILDREN OF DUNDEE.

The formal opening of the new children's ward at the Dundee Royal Infirmary has coincided with a vigorous effort to establish a cot endowment fund. Infantile mortality is engaging attention in Dundee, and the medical officer of health, Dr. Templeman, has done much to acquaint his fellow-citizens with the need for dealing by suitable organisation with its causes. Since 1883, when a children's ward was first opened, forty cots have been established, and, what is more significant of the public spirit of Dundee, each of these has been named or endowed. If present expectations be realised, Dundee should take a high place in the movement for child-welfare.

### THE EYES OF MUNITION WORKERS.

A regrettable but interesting feature of the war and general work undertaken by the Birmingham and Midland Eye Hospital has been an increase of nearly 3,000 in the number of out-patients, due, we learn, almost entirely to casualty cases among the munition workers in the city. No less than 95 per cent. of these casualties has occurred among patients engaged on the manufacture of munitions. Mr. Neville Chamberlain, the Lord Mayor, has stated that it is uncertain whether this increase has been primarily caused by the vastly larger number of persons now engaged on munition making, or by the inexperience of the newcomers. Was the number of casualties the result of accidents or merely of fatigue of the eye? Skill, of course, is required for this work, as indeed for all work. We lately heard a stone-breaker declare that he knew an employer so fond of watching his men breaking stones that his presence became a nuisance. But they got rid of him at last by breaking stones with such art that he was always hit by fragments wherever he happened to stand. The larger truth is that there is no such thing as unskilled labour.

### A CHEMIST'S TRAGIC MISTAKE.

A tragic blunder of a kind that is fortunately of very rare occurrence in this country was investigated at the Kensington Coroner's Court on March 25. Briefly, the facts are these: two women were killed by medicine for neuralgia prescribed and dispensed by a chemist, he—according to newspaper reports—having used in the preparation of the mixture strychnine in mistake for butyl chloral hydrate. The chemist, whose grief for the result of his terrible mistake can be well imagined, was apparently suffering from nerve strain due to overwork, and—not to put too fine a point on it—was not in a fit state for duties which involved the handling of potent substances. To some extent the overwork seems to have been due to a large practice as a dispenser of Insurance prescriptions, and it is unfortunate that there is no means of regulating the distribution of such prescriptions in such a way as to ensure that while one panel chemist has his thousands others in the same neighbourhood count theirs by scores. It is essential for public safety that the dispenser of medicines should be as alert as the railway signalman, and it is quite probable that this tragedy would have been avoided if this chemist's share of Insurance prescriptions had been much smaller than it was. It is difficult to imagine that a chemist possessing a sound mind in a sound body, even if he mistook the strychnine bottle for the butyl chloral hydrate bottle, would not discover his mistake before he had completed the process of compounding; for although the two drugs might appear similar to a layman, the trained eye of the pharmacist would most assuredly perceive some indication that would arouse his suspicions. There is no occasion to labour the point that the chemist had no right to prescribe, for it is obvious that the same awful mishap might have occurred had he been dispensing a proper prescription instead of encroaching on the business of a medical practitioner.

### JUVENILE DELINQUENCY.

It is an admitted fact that juvenile crime has increased since the war began, and that the recorded cases come chiefly under the categories of house-breaking, thieving, stone-throwing, and wilful damage and destruction: in other words, are chiefly crimes of indiscipline. That war influences, direct and indirect, are partly responsible for this state of things is probable enough; though the uncensored and depraved American cinema film has doubtless a strong influence in the same direction. The State Children's Association regards lack of parental control owing to absence of so many fathers abroad, darkened streets, curtailed educational facilities, and absence of social workers from boys' clubs and Church Brigades as factors tending in the same direction; and is highly concerned at certain suggestions that have been made for more repressive punishments. The Association declares that Germany is having the same trouble with the children, but that France is not; and proceeds to some dogmatic explanations of the alleged difference. It urges the importance of social service and the reclamation of the juvenile offender, wherein it will have the sympathy of all who care for the future of the nation. Recognising all this, one feels that the Association's circular is a bit mealy-mouthed in expression, for after all, if discipline has been relaxed through the absence of soldier-fathers, it is surely the State's legitimate business to see that indiscipline is checked by an adequate system of deterrent—not vindictive—punishments for misdoing.

### DISPENSERS AND MILITARY SERVICE.

The scarcity of dispensers has been very pronounced ever since the beginning of the war, but, owing to the calling up for military service of single men of military age, the scarcity seems likely to become so acute that the Insurance Commissioners have deemed it advisable to use their influence to minimise the inconvenience that would be caused by the wholesale recruiting of pharmacists and their assistants. The Commissioners have sent a circular-letter to the Insurance Committee in each area, advising them to request the military representative to obtain a short postponement of pharmacists' applications for exemption, in order to give the committee an opportunity to confer with local pharmaceutical committees and arrange proposals to place before the tribunals. An endeavour is being made to have all the pharmaceutical applications in a given area heard on the same day. Although the Commissioners are only directly interested in the pharmaceutical service in so far as it affects the supply of medicines for insured persons, they have pointed out to the committees that unless provision is made for the maintenance of dispensing facilities for the larger section of the population that does not come within the scope of the Insurance Act, the burden of work that will fall on panel chemists will be too heavy for them to bear; accordingly they advise the committees to take into consideration the whole of the pharmaceutical service in their areas. The intervention of the Insurance Commissioners is welcomed by pharmacists, who in some districts have received scant sympathy from local tribunals in respect of applications that were made before the Commissioners came to the rescue. Some of the tribunals seem to be under the impression that the supply of women pharmacists is like the widow's cruse of oil, but on the contrary pharmacists maintain that for a long time it has been practically impossible to obtain the services of a woman pharmacist. Which view is correct?

### HOSPITALS AND PUBLIC LIBRARIES.

In view of our recent account of the arrangements made between the City Librarian and the matron of Coventry and Warwickshire Hospital for the establishment of a constant supply of books for the indoor patients and staff, it is good news to know that the scheme is developing. The library has been placed in the Nurses' Home, and the first instalment of 180 books has been arranged for. As we pointed out before, such a plan depends for its success on the co-operation of public librarians with some member of the hospital staff (in this case the matron), who will take responsibility for the care of the books. Will other public librarians and other matrons please note?

### THIS WEEK'S DRUG MARKET.

A distinct improvement is reported from Norway in the results of the cod-fishing, and under the circumstances it is difficult to see how the fancy prices that have been ruling since the beginning of the season can be maintained. At the end of March 1914 cod-liver oil could be freely purchased at one-seventh the price the dealers are now quoting, and yet the quantity of new oil available in Norway to-day is not very substantially less than the quantity that was available when the oil could be purchased at a reasonable figure. Cost of labour and freight and difficulties of transport combine to increase the price at which the oil can be sold. The excellent quality of the livers this year is particularly noteworthy, and it is by no means improbable that if the weather for the remainder of the season is favourable the total yield of oil will be well above the average, and there are strong reasons to believe that hospital authorities would be well advised to postpone their purchases, for unless Germany becomes a very large buyer or something quite unexpected happens prices should be lower than they are at present. As was anticipated, quotations for tartarated soda have been raised in consequence of the high figure to which tartaric acid has advanced; seidlitz powder is also dearer. There has been a further advance in the price of citric acid. The price of opium is not so firmly maintained. Potassium chlorate is again dearer. The scarcity of potassium permanganate has become very acute, and the price is now about twenty-five times the pre-war figure.

### TO OUR READERS.

Contributions are specially invited from any of our readers to these columns. They should deal with topical subjects and news. They must be authenticated for the information of the Editor only. The minimum payment if published is 5s. There is no hard-and-fast rule as to space, but notes of about twenty lines in length are preferred.

